# Perioperative prothrombin complex concentrate and fibrinogen administration are associated with thrombotic complications after liver transplant

**DOI:** 10.3389/fmed.2022.1043674

**Published:** 2022-11-29

**Authors:** Sarah Dehne, Carlo Riede, Rosa Klotz, Anja Sander, Manuel Feisst, Uta Merle, Markus Mieth, Mohammad Golriz, Arianeb Mehrabi, Markus W. Büchler, Markus A. Weigand, Jan Larmann

**Affiliations:** ^1^Department of Anesthesiology, University Hospital Heidelberg, Heidelberg, Germany; ^2^Department of General, Visceral, and Transplantation Surgery, University Hospital Heidelberg, Heidelberg, Germany; ^3^Institute of Medical Biometry, University Hospital Heidelberg, Heidelberg, Germany; ^4^Department of Internal Medicine IV, University Hospital Heidelberg, Heidelberg, Germany

**Keywords:** coagulation factors, thrombotic complications, liver transplant, perioperative coagulation management, postoperative complications

## Abstract

**Background:**

Use of intraoperative prothrombin complex concentrates (PCC) and fibrinogen concentrate administration has been linked to thrombotic events. However, it is unknown if its use is associated with thrombotic events after liver transplant.

**Methods and analysis:**

We conducted a *post hoc* analysis of a prospectively conducted registry database study on patients who underwent liver transplant between 2004 and 2017 at Heidelberg University Hospital, Heidelberg, Germany. Univariate and multivariate analyses were used to determine the association between PCC and fibrinogen concentrate administration and thrombotic complications.

**Results:**

Data from 939 transplantations were included in the analysis. Perioperative PCC or fibrinogen administration was independently associated with the primary composite endpoint Hepatic artery thrombosis (HAT), Portal vein thrombosis (PVT), and inferior vena cava thrombosis [adjusted HR: 2.018 (1.174; 3.468), *p* = 0.011]. PCC or fibrinogen administration was associated with the secondary endpoints 30-day mortality (OR 4.225, *p* < 0.001), graft failure (OR 3.093, *p* < 0.001), intraoperative blood loss, red blood cell concentrate, fresh frozen plasma and platelet transfusion, longer hospitalization, and longer length of stay in intensive care units (ICUs) (all *p* < 0.001). PCC or fibrinogen administration were not associated with pulmonary embolism, myocardial infarction, stroke, or deep vein thrombosis within 30 days after surgery.

**Conclusion:**

A critical review of established strategies in coagulation management during liver transplantation is warranted. Perioperative caregivers should exercise caution when administering coagulation factor concentrate during liver transplant surgery. Prospective randomized controlled trials are needed to establish causality for the relationship between coagulation factors and thrombotic events in liver transplantation. Further studies should be tailored to identify patient subgroups that will likely benefit from PCC or fibrinogen administration.

## Introduction

Thrombotic events after liver transplantation are serious complications associated with an adverse outcome.

Hepatic artery thrombosis (HAT), portal vein thrombosis (PVT), and thrombosis of the inferior vena cava occur at frequencies of up to 9, 2, and 2%, respectively (1–4). HAT can further be divided into early (occurring within 1 month after liver transplantation) or late HAT events (5). Thrombotic events are associated with complications such as organ dysfunction, acute graft loss, and increased need for retransplantation, as well as leading to higher mortality rates (6–8). The underlying mechanisms are multifactorial and remain partly unclear (9). The risk factors for early HAT are the most commonly described and include intraoperative reconstruction of arteries (4, 10), pre-existing HAT, previous abdominal surgeries, duration of surgery, cold ischemia time, and female sex as well as the age of both the recipient and the donor, split liver transplantation, higher recipient/donor weight ratio, recipient/donor cytomegalovirus (CMV) mismatch, retransplantation or increased red blood cells (≥6 units), and the need for fresh frozen plasma (≥10 units) transfusion (5–7, 9, 11–13). In the past two decades, the use of recombinant coagulation factors—in particular fibrinogen and prothrombin complex concentrates (PCC)—during liver transplantation has increased. Fibrinogen is an adhesive protein essential to platelet aggregation that forms an insoluble fibrin clot in the final stage of the blood coagulation cascade (14). PCC contain the coagulation factors II, VII, IX, and X as well as the natural coagulation inhibitors protein C and protein S (15). Its administration depends on blood loss, hemostatic disturbances, current coagulation requirements, and anesthesiologist and surgeon preference. The need for fibrinogen and PCC administration can be estimated using thrombelastometry. Recombinant coagulation factor therapy has been implicated with cardiac complications (16, 17). However, whether fibrinogen and PCC administration are associated with thrombotic complications after liver transplant is unknown. Therefore, we conducted the present study to evaluate if perioperative PCC or fibrinogen administration are associated with thrombotic complications after liver transplant.

## Materials and methods

### Study design

We performed a *post hoc* analysis of a prospectively conducted registry database study (4) on adult patients who underwent orthotopic liver transplantation at our center between January 1st, 2004 and December 31st, 2017, to assess whether perioperative PCC or fibrinogen administration is associated with thrombotic complications. Patients were included if enrolled in the surgical database and anesthesia and intensive care unit (ICU) records were available. Retransplants were only included in the analysis if the date of the last known previous transplantation was available and was more than 30 days prior to the retransplantation of interest. Patients were excluded if they were retransplanted during the observation period or died during or within 24 h post-surgery.

The study protocol conformed to the principles of the Declaration of Helsinki and was approved by the local Ethics Committee of the Medical Faculty of the Ruprecht Karl University Heidelberg (S-200/2018, March 11st, 2018). This report follows the STROBE recommendations for observational studies (18).

### Procedures

Surgery was performed according to departmental standards. At our center, the standard procedure for liver transplant is the modified piggyback technique according to Belghiti using a cavo-caval side-to-side anastomosis (19). General anesthesia followed standard operating procedures. Anesthesia was induced using propofol and sufentanil or remifentanyl and was maintained as balanced anesthesia using sevoflurane or desflurane. In addition to conventional coagulation tests, rotational thrombelastometry (TEG ^®^ or ROTEM ^®^) was introduced at our center in 2002 and has been used since as a routine measure to guide fresh-frozen plasma-, coagulation factor concentrate-, and platelet concentrate-therapy during liver transplant.

### Data collection

Data for all patients who underwent orthotopic liver transplant were retrieved from our prospectively gathered database (4) and assessed for eligibility. Demographic data, weight, height, Model of End-stage Liver Disease (MELD)-score, Child-Pugh-Score, CMV status, previous abdominal surgery, retransplantations, duration of surgery, length of ICU, and hospital stay, previous myocardial infarction, coronary heart disease and its treatment, history of stroke, previous deep vein thrombosis, HAT, PVT, vena cava inferior thrombosis, and other thrombosis types were collected from the electronic patient file and the transplant surgery database. Demographic data of donors including age, weight, height, and cold ischemia time were retrieved from the transplant surgery database. Intraoperative PCC and fibrinogen administration, red blood cell concentrate, and fresh frozen plasma and platelet transfusion were collected from anesthesia records. Coagulation factor administration and transfusion up to the third postoperative day were also extracted from ICU records for each patient.

### Outcome analysis

The pre-specified primary composite endpoint was occurrence of early HAT, PVT, or inferior vena cava thrombosis within the first 30 days after liver transplant. Thrombosis was detected with the aid of ultrasound, computer tomography, or angiography and documented in the electronic patient file.

Pre-specified secondary endpoints were the individual components of the composite endpoint, 30-day mortality, graft failure defined as failure of the liver allograft that required re-LT or resulted in death of the recipient, pulmonary embolism, myocardial infarction, stroke, and deep vein thrombosis within the first 30 days after liver transplant as well as duration of hospital stay, length of ICU stay, intraoperative blood loss, red blood cell concentrate, and fresh frozen plasma and platelet transfusion.

### Statistical analysis

The patient collective was divided into two groups with regard to coagulation factor administration in the perioperative period. One group comprised all patients without coagulation factor administration (no factor group). The other group included all patients who received PCC and/or fibrinogen (factor group). One administered PCC unit corresponded to 1,000 international units PCC and fibrinogen use was measured in grams. One transfusion unit of platelet concentrates, fresh frozen plasma, or red blood cell concentrates corresponded to 200, 300, and 300 ml, respectively. The sum of all perioperative transfusions up to the third postoperative day was calculated. The primary outcome analysis was performed using the Kaplan-Meier method and groups were compared by means of the log-rank test. The primary outcome was also analyzed using Cox regression. A hazard ratio (HR) describes the independent effect of coagulation factor administration on the composite endpoint. In case of death or retransplantation during the observation period of 30 days after initial liver transplantation, patients have been censored for the specific day.

Gender, age, and BMI of both recipient and donor, previous HAT, PVT, and vena cava inferior thrombosis, number of arterial anastomoses, recipient/donor CMV mismatch, previous abdominal surgeries, duration of surgery, cold ischemia time, split liver transplantation, recipient/donor weight ratio, retransplantation, MELD- score, Child-Pugh-score, transplant priority, and transfusion of red blood cell, fresh frozen plasma, and platelet concentrates were analyzed as factors in the Cox proportional hazards model using a univariate analysis. All covariates with *p* < 0.1 in the univariate analysis as well as all significantly different baseline variables (Table 1) and perioperative blood products administered (Table 2) with significant differences between groups were included in a multivariate analysis were included in a multivariate analysis. *P* < 0.05 was considered statistically significant. In the secondary outcome analyses, individual components of the composite endpoint and mortality in the different groups were compared using a Cox regression hazard model. Pulmonary embolism, myocardial infarction, stroke, deep vein thrombosis, and graft failure were analyzed using logistic regression (Supplementary material). Categorical data were compared using the chi-square test. Continuous data were compared using the Mann-Whitney *U*-test. Statistical analyses were performed using IBM SPSS Statistics 26.0 (SPSS, Chicago, IL) and Prism 9.2.0 (GraphPad Prism Software, Inc., San Diego, CA).

**TABLE 1:** Clinical baseline characteristics of the study cohort.

Variable	Total	No factor group	Factor group	*P*-value
*n* (% of total)	939 (100.0)	363 (38.7)	576 (61.3)	
***Recipient***				
Age (y), mean ± SD	52.2 ± 10.2	52.4 ± 10.4	52.0 ± 10.1	0.571
Male sex, *n* (%)	636 (67.7)	253 (69.7)	383 (66.5)	0.306
Height (cm), mean ± SD	173.0 ± 8.9	172.6 ± 8.8	173.3 ± 8.9	0.300
Weight (kg), mean ± SD	79.2 ± 16.7	76.6 ± 16.4	80.9 ± 16.6	**<0.001**
BMI (kg/m^2^), mean ± SD	26.4 ± 4.8	25.6 ± 4.7	26.9 ± 4.8	**<0.001**
Retransplantation, *n* (%)	81 (8.6)	19 (5.2)	62 (10.8)	**0.003**
**Child-Pugh, *n* (%)**				
A	266 (28.3)	167 (46.0)	99 (17.2)	**<0.001**
B	248 (26.4)	91 (25.1)	157 (27.3)	0.459
C	425 (45.3)	105 (28.9)	320 (55.6)	**<0.001**
MELD, mean ± SD	19.3 ± 10.4	14.3 ± 7.8	22.5 ± 10.7	**<0.001**
***Donor***				
Age (y), mean ± SD	57.6 ± 17.5	56.6 ± 17.9	58.2 ± 17.2	0.209
Male sex, *n* (%)	493 (52.5)	172 (47.4)	321 (55.7)	**0.013**
Height (cm), mean ± SD	171.5 ± 10.9	171.1 ± 9.7	171.7 ± 11.7	0.171
Weight (kg), mean ± SD	77.0 ± 15.2	74.3 ± 14.4	78.7 ± 15.4	**<0.001**
BMI (kg/m^2^), mean ± SD	26.1 ± 5.8	25.2 ± 3.8	26.7 ± 6.7	**<0.001**
Weight ratio recipient/donor, mean ± SD	1.05 ± 0.24	1.05 ± 0.20	1.05 ± 0.26	0.853
***Urgency, n (%)***				
T	880 (93.7)	350 (96.4)	530 (92.0)	**0.007**
HU	59 (6.3)	13 (3.6)	46 (8.0)	**0.007**

Data are presented as mean ± SD, or as absolute number (percentage). *P*-values refer to the comparison between the no factor and factor groups. Continuous data were compared using the Mann-Whitney *U*-test. Categorical variables were compared using the chi-square test. Bold face indicates *p*-values < 0.05. SD, standard deviation; BMI, body mass index; MELD, Model for End-stage Liver Disease; T, transplantable; HU, high urgency.

**TABLE 2:** Outcome analysis.

Variable	Total	No factor group	Factor group	*P*-value
Primary endpoint, *n* (%)	89 (9.5) *n* = 939	24 (6.6) *n* = 363	65 (11.3) *n* = 576	**0.017**
Hepatic artery thrombosis, *n* (%)	52 (5.5) *n* = 939	15 (4.1) *n* = 363	37 (6.4) *n* = 576	0.135
Portal vein thrombosis, *n* (%)	30 (3.2) *n* = 939	6 (1.7) *n* = 363	24 (4.2) *n* = 576	**0.033**
Thrombosis of the inferior vena cava, *n* (%)	16 (1.7) *n* = 939	4 (1.1) *n* = 363	12 (2.1) *n* = 576	0.258
30-day mortality, *n* (%)	67 (7.1) *n* = 939	9 (2.5) *n* = 363	58 (10.1) *n* = 576	**<0.001**
Graft failure, *n* (%)	108 (11.5) *n* = 939	20 (5.5) *n* = 363	88 (15.3) *n* = 576	**<0.001**
Pulmonary embolism, *n* (%)	17 (1.8) *n* = 939	5 (1.4) *n* = 363	12 (2.1) *n* = 576	0.429
Myocardial infarction, *n* (%)	20 (2.1) *n* = 939	6 (1.7) *n* = 363	14 (2.4) *n* = 576	0.422
Stroke, *n* (%)	7 (0.7) *n* = 939	1 (0.3) *n* = 363	6 (1.0) *n* = 576	0.184
Deep vein thrombosis, *n* (%)	5 (0.5) *n* = 939	1 (0.3) *n* = 363	4 (0.7) *n* = 576	0.390
Length of intensive care unit stay (d), mean ± SD	9.6 ± 20.2 *n* = 934	5.9 ± 11.1 *n* = 361	12.0 ± 23.9 *n* = 573	**<0.001**
Length of hospital stay (d), mean ± SD	48.7 ± 40.2 *n* = 939	38.9 ± 30.3 *n* = 363	55.0 ± 44.3 *n* = 576	**<0.001**
Intraoperative blood loss (ml), mean ± SD	4370.9 ± 4490.7 *n* = 781	2793.6 ± 2559.4 *n* = 282	5262.3 ± 5068.0 *n* = 499	**<0.001**
Transfused RBC (TU), mean ± SD	9.2 ± 11.3 *n* = 939	4.2 ± 5.4 *n* = 363	12.3 ± 12.8 *n* = 576	**<0.001**
Transfused PLT (TU), mean ± SD	4.0 ± 5.0 *n* = 939	1.6 ± 2.3 *n* = 363	5.5 ± 5.7 *n* = 576	**<0.001**
Transfused FFP (TU), mean ± SD	20.22 ± 17.25 *n* = 939	13.62 ± 10.96 *n* = 363	24.37 ± 19.11 *n* = 576	**<0.001**

Data are presented as absolute number (percentage) or mean ± SD. *P*-values refer to the comparison between no factor and factor group. Categorial data were compared using the chi-square test. Continuous data were compared using the Mann-Whitney *U*-test. Bold face indicates *p*-values < 0.05. SD, standard deviation; RBC, Red blood cells; PLT, Platelet concentrates; FFP, Fresh frozen plasma; TU, Transfusion units.

## Results

**FIGURE 1:**
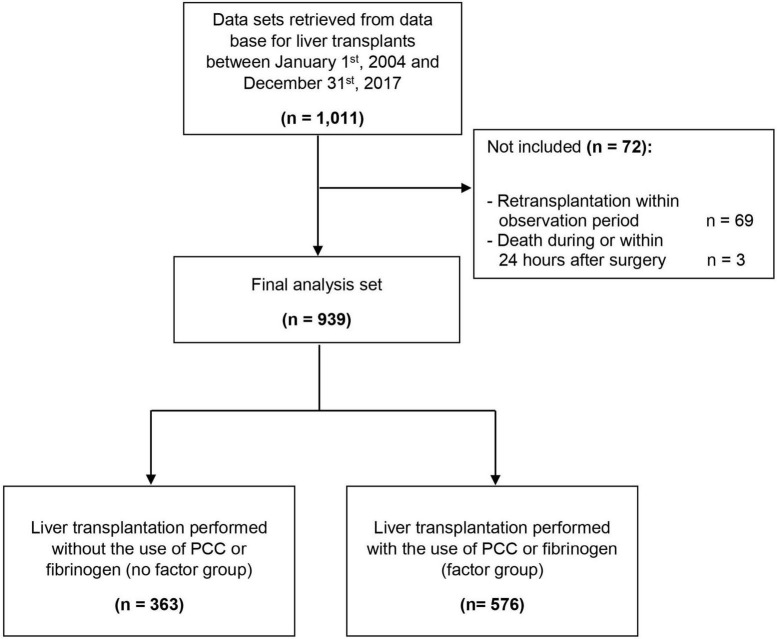
Participant flow chart. PCC, prothrombin complex concentrate.

We extracted 1,011 data sets of patients who underwent orthotopic liver transplant between 2004 and 2017 from the registry. 72 patients were not enrolled because of retransplantation during the observation period or death during or 24 h after surgery. The final analysis set consisted of 939 patients (Figure 1).

### Donor and recipient characteristics

Main clinical and demographic baseline characteristics are presented in Table 1 and Supplementary Table 1. Mean age was 52.2 ± 10.2 years. A total of 67.7% of the participants were male. Average weight was 79.2 ± 16.7 kg with a BMI of 26.4 ± 4.8 kg/m^2^. MELD-score among the recipients was 19.3 ± 10.4. The most common Child-Pugh-Score was C (45.3%). Pre-existing conditions included coronary heart disease in 114 (12.1%) patients, myocardial infarction in 23 (2.4%), and stroke in 9 (1%). HAT, PVT, and thrombosis of the inferior vena cava were reported for 13 (1.4%), 98 (10.4%), and 2 patients (0.2%), respectively. Previous deep vein thrombosis was seen in 19 (2%) participants and 119 patients had another thrombotic event (12.7%). 326 patients had had prior surgery (34.7%) and 81 underwent retransplantation (8.6%). Mean donor age was 57.6 ± 17.5 years, with a weight of 77.0 ± 15.2 kg and a BMI of 26.1 ± 5.8 kg/m^2^. Recipient/donor weight ratio was 1.05 ± 0.24. CMV-mismatch was reported in 235 patients (25%). In most transplant surgeries, one arterial anastomosis was performed (84.3%). Cold ischemia time was 8.6 ± 3.1 h and surgery time was 5.8 ± 1.5 h on average. Duration of hospital stay was 48.7 ± 40.2 days.

The no factor group comprised 363 patients and the factor group contained 576 patients. Patients in the factor group received 3,350 ± 3,500 units PCC and 4.59 ± 4.6 g fibrinogen on average. Patients in both groups were given fresh frozen plasma [334 (92.0%) vs. 553 (96.0%) in the no factor vs. factor group, *p* = 0.009]. The average weight and BMI were higher in the factor group. However, this is reflected in the donor data. Consequently, the recipient/donor weight ratio was not significantly different. Donors were more frequently male in the factor group. Between groups, there were no differences regarding recipient age, gender, and height and donor age and height, or in cold ischemic time, number of arterial anastomoses, and recipient/donor CMV mismatch. Past medical history of recipients was similar for both groups. However, there were more retransplantations in the factor group. The MELD-score was higher, the most common Child-Pugh-Score was C, more patients were registered as high urgency for transplant, and the average duration of surgery and length of hospital stay were longer in the factor group.

### Association with hepatic artery thrombosis, portal vein thrombosis, and inferior vena cava thrombosis

**FIGURE 2:**
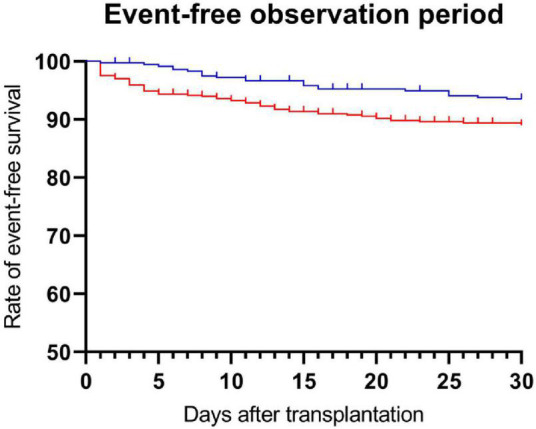
Administration of recombinant coagulation factors and event-free survival. Patients were divided into no factor and factor groups. The *p*-value was evaluated using the log-rank test (*p* = 0.01). (Blue), no factor group; (Red), factor group.

In total, 89 (9.5%) patients met the primary endpoint within the first 30 days after liver transplantation. The composite of HAT, PVT, or inferior vena cava thrombosis was more frequent in patients who received coagulation factor concentrates [24 (6.6%) vs. 65 (11.3%) patients, no factor group vs. factor group, *p* = 0.017, Table 2)]. Kaplan-Meier analysis revealed a significant benefit for patients who did not receive coagulation factor concentrates (log rank test: *p* = 0.01, Figure 2).

Univariate predictors of the primary endpoint are listed in Table 3. Eight variables with *p* < 0.100 were identified and integrated into the multivariate cox regression model: administration of recombinant coagulation factors, recipient age and gender, donor age, recipient/donor weight ratio, split liver transplantation, previous PVT, and surgery time. Baseline variables (donor gender, BMI of donor and recipient, retransplantations, urgency, MELD-Score, and Child-Pugh-Score) as well as perioperative blood products (transfusion of red blood cells (RBC), fresh frozen plasma, and platelets), all of which were significantly different between groups, were included into the multivariate cox regression model.

**TABLE 3:** Proportional hazards regression on primary endpoint.

	Univariate		Multivariate	
		
Variable	HR [95% CI]	*P*-value	Adjusted HR [95% CI]	*P*-value
Age recipient	0.983 [0.964; 1.002]	0.076	0.989 [0.968; 1.010]	0.296
Age donor	0.987 [0.976; 0.998]	0.021	0.992 [0.979; 1.006]	0.265
Male recipient	0.644 [0.422; 0.981]	0.041	1.332 [0.832; 2.132]	0.233
Male donor	0.736 [0.485; 1.118]	0.151	1.272 [0.798; 2.029]	0.312
BMI recipient	0.981 [0.939; 1.026]	0.412	0.999 [0.951; 1.050]	0.970
BMI donor	1.016 [0.985; 1.048]	0.304	**1.024 [1.000; 1.047]**	**0.045**
Weight ratio recipient/donor	2.039 [1.195; 3.480]	0.009	1.657 [0.922; 2.978]	0.091
CMV-mismatch	0.657 [0.382; 1.128]	0.127		
Cold ischemia time	1.030 [0.959; 1.106]	0.411		
History of abdominal surgery	1.090 [0.709; 1.676]	0.695		
Retransplantation	1.206 [0.605; 2.402]	0.594	1.275 [0.603; 2.694]	0.525
Split liver transplantation	3.117 [1.762; 5.516]	< 0.001	**2.339 [1.171; 4.673]**	**0.016**
Previous HAT	1.880 [0.463; 7.641]	0.377		
Previous PVT	2.330 [1.389; 3.908]	0.001	**2.140 [1.231; 3.721]**	**0.007**
Previous thrombosis of the inferior vena cava	0.050 [0.000;66893781.3]	0.779		
Surgery time	1.289 [1.141; 1.455]	<0.001	**1.217 [1.059; 1.399]**	**0.006**
Number of arterial anastomosis	1.294 [0.855; 1.957]	0.223		
Administration of recombinant coagulation factors	1.834 [1.148; 2.929]	0.011	**2.018 [1.174; 3.468]**	**0.011**
No RBC *(reference)*		0.921		0.432
RBC (1-5 TU)	1.037 [0.554; 1.941]	0.910	0.801 [0.389; 1.648]	0.546
RBC (≥ 6 TU)	1.112 [0.621; 1.992]	0.721	0.592 [0.256; 1.371]	0.221
No FFP *(reference)*		0.503		0.530
FFP (1-9 TU)	1.029 [0.348; 3.040]	0.959	0.967 [0.312; 3.000]	0.953
FFP (≥10 TU)	1.365 [0.498; 3.744]	0.545	1.381 [0.431; 4.426]	0.587
Platelets (TU)	1.021 [0.983; 1.059]	0.287	1.009 [0.960; 1.060]	0.723
MELD	0.991 [0.970; 1.011]	0.371	0.979 [0.946; 1.013]	0.228
***Child-Pugh***				
A *(reference)*		0.436		0.987
B	1.177 [0.690; 2.007]	0.550	0.954 [0.517; 1.762]	0.881
C	0.849 [0.511; 1.411]	0.528	0.986 [0.460; 2.114]	0.972
***Urgency***				
HU	0.711 [0.261; 1.937]	0.505	0.870 [0.286; 2.641]	0.805

HR estimated from the proportional hazards analysis were reported with corresponding 95% CIs. Bold face indicates *p*-values < 0.05 in multivariable proportional hazards analysis. HR, Hazard ratio; CI, confidence interval; BMI, body mass index; HAT, hepatic artery thrombosis; PVT, portal vein thrombosis; RBC, red blood cells; FFP, fresh frozen plasma; MELD, Model for End-stage Liver Disease; CMV, cytomegalovirus T, transplantable; HU, high urgency.

### Independent predictors for hepatic artery thrombosis, portal vein thrombosis, and inferior vena cava thrombosis

In the multivariate analysis, administration of recombinant coagulation factors remained independently associated with the primary composite endpoint [adjusted HR: 2.018 (1.174; 3.468), *p* = 0.011]. Other variables significantly associated with the primary endpoint were: donor BMI [adjusted HR: 1.024 (1.000; 1.047), *p* = 0.045], split liver transplantation [adjusted HR: 2.339 (1.171; 4.673), *p* = 0.016], previous PVT [adjusted HR: 2.140 (1.231; 3.721), *p* = 0.007], and duration of surgery [adjusted HR 1.217 (1.059; 1.399), *p* = 0.006].

### Secondary endpoints

Of the individual components in the composite endpoint, PVT occurred more often in the factor group (1.7% vs. 4.2%, no factor group vs. factor group, *p* = 0.033) (Table 2). There was no difference in the rate of HAT and thrombosis of the inferior vena cava (HAT: 4.1% vs. 6.4%, *p* = 0.135; thrombosis of the vena cava inferior: 1.1% vs. 2.1%, *p* = 0.258, no factor group vs. factor group) (Table 2).

In total, 67 patients (7.1%) died within 30 days post-surgery. We found an association between coagulation factor administration and 30-day mortality after liver transplant (2.5% vs. 10.1%, no factor group vs. factor group, *p* < 0.001) (Table 2). Also, graft failure was more likely in patients who received coagulation factors (5.5% vs. 15.3%, no factor group vs. factor group, *p* < 0.001) (Table 2). Pulmonary embolism, myocardial infarction, stroke, and deep vein thrombosis were not associated with coagulation factor administration. Intraoperative blood loss (2,794 ± 2,559 ml vs. 5,262 ± 5,068 ml, no factor group vs. factor group, *p* < 0.001) as well as red blood cell concentrate (4.2 ± 5.4 units vs. 12.3 ± 12.8 units, no factor group vs. factor group, *p* < 0.001), platelet transfusion (1.6 ± 2.3 units vs. 5.5 ± 5.7 units, no factor group vs. factor group, *p* < 0.001) and fresh frozen plasma transfusion (13.62 ± 10.96 units vs. 24.37 ± 19.11 units, no factor group vs. factor group, *p* < 0.001) were higher with coagulation factor administration (Table 2). Length of hospital stay (55.0 ± 44.3 days vs. 38.9 ± 30.3 days, *p* < 0.001) and length of ICU stay (12.0 ± 23.9 days vs. 5.9 ± 11.1 days, *p* < 0.001) were longer in the factor group (Table 2). In the regression analysis, administration of PCC or fibrinogen was also associated with 30-day mortality [HR 4.225 (2.093; 8.527), *p* < 0.001], graft failure [OR 3.093 (1.867; 5.123), *p* < 0.001] and PVT [HR 2.650 (1.083; 6.483), *p* = 0.033] (Supplementary Table 2).

## Discussion

We report that perioperative PCC or fibrinogen administration was independently associated with the composite endpoint defined as new onset of early HAT, PVT, and/or inferior vena cava thrombosis within the first 30 days after liver transplant.

Donor BMI, previous PVT, split liver transplantation, and surgery time were additional independent risk factors. Furthermore, PCC or fibrinogen administration during liver transplant surgery was associated with the pre-specified secondary endpoints 30-day mortality, graft failure, intraoperative blood loss, red blood cell concentrate transfusion, platelet transfusion, duration of hospital stay, and length of ICU stay. There was no significant between-group difference regarding pulmonary embolism, myocardial infarction, stroke, or deep vein thrombosis.

Thus far, several studies have examined risk factors for thrombotic events after liver transplant, in particular HAT (4). In line with our findings, split liver transplant, previous PVT, and surgery time have been described as risk factors (5–7, 9, 11–13, 20, 21). One multivariate analysis has found an association between increased blood transfusion and the occurrence of HAT (12). However, in line with our findings, other researchers have demonstrated that increased intraoperative transfusion was not an independent risk factor for HAT (5, 6).

Hemostasis in patients with end-stage liver disease varies with the underlying disease. It is characterized by reduced synthesis capacity of coagulation and anticoagulation factors as well as fibrinolytic and antifibrinolytic factors (22, 23). An imbalance of these factors can lead to thrombosis and bleeding (24, 25). Severe blood loss may occur, especially during liver transplant. Currently, there is no transfusion guideline for liver transplant (26). Traditionally, fresh frozen plasma has been used to correct coagulopathy when intraoperative bleeding occurs during liver transplant. Research has demonstrated an association between the amount of blood product transfused and mortality, as well as further adverse advents like ICU readmission, after liver transplant (22, 26, 27). The establishment of viscoelastic methods like thrombelastography made the targeted administration of coagulation factor concentrates, platelets, plasma, and fibrinolysis inhibitors during liver transplantation possible (27). Research has shown that viscoelastic coagulation management algorithms guiding therapy with fibrinogen concentrate and PCC can lead to a reduction in the transfusion requirements for fresh frozen plasma, red blood cells, and platelets as well as a reduced incidence of massive transfusion (28). We did not observe a reduction in transfusion requirements associated with factor administration, which is likely due to the factor group suffering from more advanced liver failure.

However, there is evidence that recombinant coagulation factor therapy is associated with cardiac complications, venous thromboembolism, disseminated intravascular coagulation, and microvascular thrombosis outside of transplant surgery (16, 17, 29). Current evidence for coagulation factor administration associated with thrombotic events after liver transplantation is sparse. So far, perioperative fibrinogen or PCC administration during liver transplant is considered to be safe (30). Kirchner et al. examined treatment with fibrinogen concentrate or PCC in 266 patients undergoing liver transplant (31). In contrast to our findings, there were no significant differences in HAT or PVT occurrence between the fibrinogen concentrate or PCC group and the non-coagulation factor concentrates group (31). In a retrospective analysis, Srivastava et al. studied the administration of PCC in 262 patients undergoing liver transplantation. After propensity score−matching, the use of PCC was associated with a lower transfusion requirement. In addition, no thromboembolic complications associated with PCC were observed. However, it is notable that no thromboses were diagnosed in the entire patient population under investigation (32). Due to the relatively small case number and short observation period of only 7 and 10 days (31, 32), these results are not directly comparable to our findings. The authors of a narrative review based on nine observational studies and expert opinion concluded that deranged hemostasis can be restored in the majority of liver transplant patients using point-of-care coagulation measurement-guided fibrinogen concentrate and PCC administration (33). However, they postulate that plasma transfusion is required in a share of patients undergoing liver transplantation (33) and added that all measures carry a risk for thromboembolic complications and therefore should be used with caution and following institutional protocols (33).

As defined by our standard of care, coagulation factor administration was controlled by viscoelastic point-of-care methods. By performing viscoelastic point-of-care methods and determining common laboratory parameters such as fibrinogen, International Normalized Ratio, or partial thromboplastin time, only deficits in procoagulant factors or antifibrinolytic factors are measured (34). In contrast, anticoagulant factors are not captured. Unlike transfusion of fresh frozen plasma, factor-based coagulation management does not replace anticoagulant factors. Thus, there might be an imbalance between pro- and anticoagulation, potentially in favor of thrombotic complications.

Our study has some limitations that need to be addressed. Since this is a single center study, the generalizability and external validity of the results may be limited. There is an unequal distribution of baseline values between the groups. As a result, patients with fibrinogen or PCC administration had a worse liver function preoperatively (higher MELD-score, Child-Pugh-Score, number of retransplantations, and high urgency listing). Average weight, BMI of the donor and recipient and duration of surgery were also higher in patients with fibrinogen or PCC administration. All factors except for duration of surgery and BMI of the donor, however, were ruled out as independent risk factors in our primary outcome analysis. The number of included individuals was limited by the availability of digitalized anesthesia and ICU records, and only patients recruited in the transplant surgical databases could be analyzed. Furthermore, ROTEM results were not available and could not be reviewed. Due to the division of patients into two groups, the quantity of coagulation factor administration was not considered.

In conclusion, we demonstrated that perioperative PCC or fibrinogen administration is independently associated with the primary composite endpoint, HAT, PVT, and inferior vena cava thrombosis. PCC or fibrinogen administration was associated with 30-day mortality after liver transplant, graft failure, intraoperative blood loss, red blood cell concentrate and fresh frozen plasma transfusion, platelet transfusion, length of hospital stay, and length of ICU stay. The liberal use of PCC or fibrinogen during liver transplant must be critically reviewed and reconsidered. These findings will be instrumental for designing prospective studies delineating the effects of coagulation factor administration on thrombotic complications among patients undergoing liver transplant.

## Data availability statement

The raw data supporting the conclusions of this article will be made available by the authors, without undue reservation.

## Ethics statement

The studies involving human participants were reviewed and approved by the Ethics Committee of the Medical Faculty of the Ruprecht Karl University Heidelberg (S-200/2018, March 11st, 2018). Written informed consent for participation was not required for this study in accordance with the national legislation and the institutional requirements.

## Author contributions

SD, CR, RK, and JL: design research and study protocol. SD, CR, AS, and JL: formal analysis. AS and MF: methodology and statistical consultation. SD, CR, RK, MG, MM, UM, AM, MB, and MW: data curation. SD, CR, and JL: write the first draft of the manuscript. All authors critically reviewed and revised the manuscript and approved the final work.

## Abbreviations

BMI: Body mass index
CMV: Cytomegalovirus
FFP: Fresh Frozen Plasma
HR: Hazard Ratio
HAT: Hepatic artery thrombosis
HU: High urgency
ICU: Intensive care unit
MELD: Model of End-stage Liver Disease
PVT: Portal vein thrombosis
PCC: Prothrombin complex concentrates
T: Transplantable
TEG^®^ or ROTEM^®^: Rotational thrombelastometry
RBC: Red blood cells.
