# Supplementary material for: Perioperative prothrombin complex concentrate and fibrinogen administration are associated with thrombotic complications after liver transplant
Source: Front Med (Lausanne). 2022 Nov 29;9:1043674. doi: 10.3389/fmed.2022.1043674 (PMC9745140; doi:10.3389/fmed.2022.1043674)
Supplement: Supplementary file 1 [file Data_Sheet_1.docx]

**Supplemental Material**

**Supplemental Table 1: Medical history and surgery data of the study cohort**

| ***Variable*** | **Total** | **No factor group** | **Factor group** | **p-value** |
| --- | --- | --- | --- | --- |
| ***Medical history, n (%)*** |  |  |  |  |
| Coronary heart disease | 136 (14.5) | 46 (12.7) | 90 (15.6) | .211 |
| Without intervention | 78 (8.3) | 30 (8.3) | 48 (8.3) | .970 |
| With PCI | 25 (2.7) | 6 (1.7) | 19 (3.3) | .127 |
| With CABG | 10 (1.1) | 3 (0.8) | 7 (1.2) | .572 |
| Myocardial infarction | 23 (2.4) | 7 (1.9) | 16 (2.8) | .412 |
| Stroke | 9 (1.0) | 3 (0.8) | 6 (1.0) | .742 |
| Previous deep vein thrombosis | 19 (2.0) | 12 (3.3) | 7 (1.2) | .027 |
| Previous hepatic artery thrombosis | 13 (1.4) | 3 (0.8) | 10 (1.7) | .245 |
| Previous portal vein thrombosis | 98 (10.4) | 39 (10.7) | 59 (10.2) | .807 |
| Previous thrombosis of the inferior vena cava | 2 (0.2) | 1 (0.3) | 1 (0.2) | .742 |
| Other thrombosis in history | 119 (12.7) | 41 (11.3) | 78 (13.5) | .668 |
| Previous abdominal surgery, n (%) | 326 (34.7) | 132 (36.4) | 194 (33.7) | .400 |
| ***Surgery data*** |  |  |  |  |
| Cold ischemia time (h), mean ± SD | 8.6 ± 3.1 | 8.6 ± 2.8 | 8.6 ± 3.2 | .165 |
| Surgery time (h), mean ± SD | 5.8 ± 1.5 | 5.4 ± 1.5 | 6.0 ± 1.5 | **<.001** |
| CMV-Mismatch, n (%) | 235 (25.0) | 88 (24.2) | 147 (25.5) | .660 |
| Number of arterial anastomosis, n (%) |  |  |  |  |
| 1 | 792 (84.3) | 306 (84.3) | 486 (84.4) | .975 |
| 2 | 135 (14.4) | 51 (14.0) | 84 (14.6) | .820 |
| ≥ 2 | 12 (1.3) | 6 (1.7) | 6 (1.0) | .417 |

Data are presented as mean ± SD, or as absolute number (percentage). P-values refer to the comparison between the no factor and factor groups. Continuous data were compared using the Mann-Whitney U test. Categorical variables were compared using the chi-square test. Bold face indicates p-values < .05. SD, standard deviation; PCI, Percutaneous coronary intervention; CABG, Coronary Artery Bypass Graft; RBC, red blood cell; CMV, cytomegalovirus.

**Supplemental Table 2: Regression analysis**

| **Dependent variable** | **HR [95% CI]** | **p-value** |
| --- | --- | --- |
| Hepatic artery thrombosis | 1.663 [0.913; 3.030] | .097 |
| Portal vein thrombosis | **2.650** **[1.083; 6.483]** | **.033** |
| Thrombosis of the inferior vena cava | 1.976 [0.637; 6.128] | .238 |
| 30-day mortality | **4.225 [****2.093; 8.527]** | **<.001** |
| **Dependent variable** | **OR [95% CI]** | **p-value** |
| Graft failure | **3.093 [****1.867; 5.123]** | **<.001** |
| Pulmonary embolism | 1.523 [0.532; 4.361] | .433 |
| Myocardial infarction | 1.482 [0.564; 3.893] | .424 |
| Stroke | 3.811 [0.457; 31.781] | .216 |
| Deep vein thrombosis | 2.531 [0.282; 22.738] | .407 |

HR respectively OR estimated from proportional hazards or logistic analysis were reported with corresponding 95% CIs. Data were compared using proportional hazards regression for the variables hepatic artery thrombosis, portal vein thrombosis, thrombosis of the inferior vena cava, and 30-day mortality. Logistics regression was used for the variables graft failure, pulmonary embolism, myocardial infarction, stroke, and deep vein thrombosis. P-values refer to the comparison of the respective endpoint between no factor and factor group. Bold face indicates p-values <.05.
